# Flow-induced Shear Stress Confers Resistance to Carboplatin in an Adherent Three-Dimensional Model for Ovarian Cancer: A Role for EGFR-Targeted Photoimmunotherapy Informed by Physical Stress

**DOI:** 10.3390/jcm9040924

**Published:** 2020-03-28

**Authors:** Shubhankar Nath, Michael Pigula, Amjad P. Khan, William Hanna, Mustafa Kemal Ruhi, Farzaneh Mahmoodpoor Dehkordy, Karthik Pushpavanam, Kaushal Rege, Kaitlin Moore, Yujiro Tsujita, Christina Conrad, Fatih Inci, Marcela G. del Carmen, Walfre Franco, Jonathan P. Celli, Utkan Demirci, Tayyaba Hasan, Huang-Chiao Huang, Imran Rizvi

**Affiliations:** 1 Wellman Center for Photomedicine, Massachusetts General Hospital, Harvard Medical School, Boston, MA 02114, USA; snath.vet2000@gmail.com (S.N.); m.pigula94@gmail.com (M.P.); APKHAN@mgh.harvard.edu (A.P.K.); mkruhi@email.unc.edu (M.K.R.); FMahmoodpoorDehkordy@mgh.harvard.edu (F.M.D.); kmoore31@mgh.harvard.edu (K.M.); yujirotsujita@gmail.com (Y.T.); wfranco@mgh.harvard.edu (W.F.); thasan@mgh.harvard.edu (T.H.); 2 Department of Physics, College of Science and Mathematics, University of Massachusetts at Boston, Boston, MA 02125, USA; william.hanna001@gmail.com (W.H.); jonathan.celli@umb.edu (J.P.C.); 3 Joint Department of Biomedical Engineering, University of North Carolina at Chapel Hill, Chapel Hill, NC and North Carolina State University, Raleigh, NC 27599, USA; 4 School for Engineering of Matter, Transport and Energy, Ira A. Fulton Schools of Engineering, Arizona State University, Tempe, AZ 85287, USA; kpsubram@asu.edu (K.P.); Kaushal.Rege@asu.edu (K.R.); 5 Department of Urology, National Defense Medical College, Tokorozawa, Saitama 359-8513, Japan; 6 Fischell Department of Bioengineering, University of Maryland, College Park, MD 20742, USA; cconrad8@terpmail.umd.edu (C.C.); hchuang@umd.edu (H.-C.H.); 7 Bio-Acoustic MEMS in Medicine (BAMM) Laboratory, Canary Center at Stanford for Cancer Early Detection, Department of Radiology School of Medicine Stanford University, Palo Alto, CA 94304, USA; finci@stanford.edu (F.I.); utkan@stanford.edu (U.D.); 8 Division of Gynecologic Oncology, Vincent Obstetrics and Gynecology, Massachusetts General Hospital, Harvard Medical School, Boston, MA 02114, USA; mdelcarmen@mgh.harvard.edu; 9 Marlene and Stewart Greenebaum Cancer Center, University of Maryland School of Medicine, Baltimore, MD 21201, USA; 10 Lineberger Comprehensive Cancer Center, University of North Carolina School of Medicine, Chapel Hill, NC 27599, USA

**Keywords:** ovarian cancer, epidermal growth factor receptor (EGFR), mitogen-activated protein kinase/extracellular signal-regulated kinase (MEK), extracellular signal-regulated kinase (ERK), chemoresistance, fluid shear stress, ascites, perfusion model, photoimmunotherapy (PIT), photodynamic therapy (PDT), carboplatin

## Abstract

A key reason for the persistently grim statistics associated with metastatic ovarian cancer is resistance to conventional agents, including platinum-based chemotherapies. A major source of treatment failure is the high degree of genetic and molecular heterogeneity, which results from significant underlying genomic instability, as well as stromal and physical cues in the microenvironment. Ovarian cancer commonly disseminates via transcoelomic routes to distant sites, which is associated with the frequent production of malignant ascites, as well as the poorest prognosis. In addition to providing a cell and protein-rich environment for cancer growth and progression, ascitic fluid also confers physical stress on tumors. An understudied area in ovarian cancer research is the impact of fluid shear stress on treatment failure. Here, we investigate the effect of fluid shear stress on response to platinum-based chemotherapy and the modulation of molecular pathways associated with aggressive disease in a perfusion model for adherent 3D ovarian cancer nodules. Resistance to carboplatin is observed under flow with a concomitant increase in the expression and activation of the epidermal growth factor receptor (EGFR) as well as downstream signaling members mitogen-activated protein kinase/extracellular signal-regulated kinase (MEK) and extracellular signal-regulated kinase (ERK). The uptake of platinum by the 3D ovarian cancer nodules was significantly higher in flow cultures compared to static cultures. A downregulation of phospho-focal adhesion kinase (p-FAK), vinculin, and phospho-paxillin was observed following carboplatin treatment in both flow and static cultures. Interestingly, low-dose anti-EGFR photoimmunotherapy (PIT), a targeted photochemical modality, was found to be equally effective in ovarian tumors grown under flow and static conditions. These findings highlight the need to further develop PIT-based combinations that target the EGFR, and sensitize ovarian cancers to chemotherapy in the context of flow-induced shear stress.

## 1. Introduction

**Figure 1:**
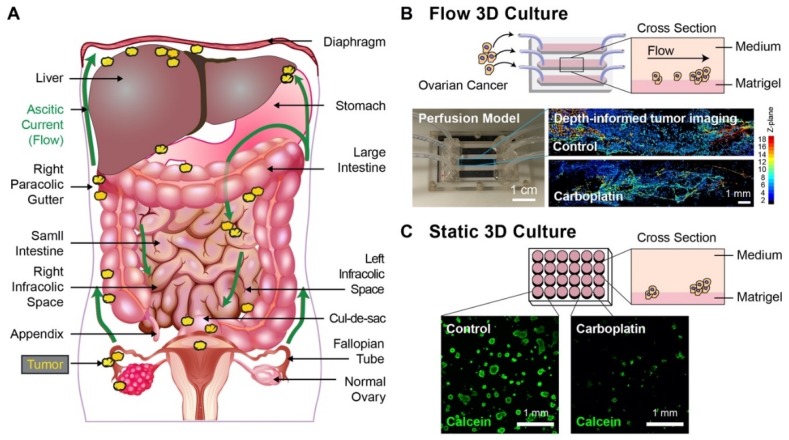
(**A**) A schematic of ovarian cancer metastases involving tumor cells or clusters (yellow) shedding from a primary site and disseminating along ascitic currents of peritoneal fluid (green arrows) in the abdominal cavity. Ovarian cancer typically disseminates in four common abdomino-pelvic sites: (1) cul-de-sac (an extension of the peritoneal cavity between the rectum and back wall of the uterus); (2) right infracolic space (the apex formed by the termination of the small intestine of the small bowel mesentery at the ileocecal junction); (3) left infracolic space (superior site of the sigmoid colon); (4) Right paracolic gutter (communication between the upper and lower abdomen defined by the ascending colon and peritoneal wall). (**B**) The schematic of a perfusion model used to study the impact of sustained fluid flow on treatment resistance and molecular features of 3D ovarian cancer nodules (Top left). A side view of the perfusion model and growth of ovarian cancer nodules to a stromal bed (Top right). The photograph of a perfusion model used in the experiments (Bottom left) and depth-informed confocal imaging of ovarian cancer nodules in channels with and without carboplatin treatment (Bottom right). The perfusion model is 24 × 40 mm, with three channels that are 4 × 30 mm each and a height of 254 μm. The inlet and outlet ports of channels are 2.2 mm in diameter and positioned 5 mm from the edge of the chip. (**C**) A schematic of a 24-well plate model used to study the treatment resistance and molecular features of 3D ovarian cancer nodules under static conditions (without flow) (Top left). A side view of the static models and growth of ovarian cancer nodules on a stromal bed (Top right). Confocal imaging of 3D ovarian cancer nodules in a 24-well plate without and with carboplatin treatment (Bottom). Scale bars: 1 mm.

Advanced-stage epithelial ovarian cancer is the leading cause of death from gynecologic malignancies in the United States, with a five-year survival rate of only 30% [1], a grim statistic that has remained unchanged for decades. Surgical debulking and platinum-based chemotherapy remain the standard of care for advanced stage disease. While most patients with metastatic ovarian cancer will initially respond to treatment, the vast majority (~75%) will develop recurrent disease within two years of their initial diagnosis. Recurrent ovarian cancer is incurable [2]. Significant efforts have been made to understand the inherent and acquired mechanisms of platinum-resistance, but meaningful improvements in the platinum-free interval, the time between the last cycle of platinum and disease progression, and in sustained survival benefits remain elusive [2]. Progression of ovarian cancer is often associated with an increased production and retention of fluid (ascites) in the peritoneum (Figure 1A). The role of peritoneal flow-induced shear stress on chemoresistance in metastatic ovarian cancer, however, remains poorly understood. Here, we investigate, for the first time, the influence of flow-induced shear stress on carboplatin resistance in a perfusion model for three-dimensional (3D) ovarian cancer (Figure 1B).

Ovarian cancer disseminates as individual cells or cell-clusters that exfoliate from the primary tumor and spread via transcoelomic, lymphatic, or hematogenous routes to distant sites [3,4,5,6,7,8,9,10]. Among these routes, transcoelomic metastases are the most common and are associated with the frequent production of malignant ascites as well as the highest morbidity and mortality rates [3,4]. Malignant ascites provide fluid-based routes for exfoliated tumor clusters and cells to spread throughout the peritoneum, as well as a nutrient- and chemokine-rich environment to promote the growth and motility of cancer cells [3,4,5]. Establishment of metastatic colonies in the peritoneum has a significant negative impact on survival outcomes and makes the disease exceptionally difficult to treat, with frequent recurrence [2,8,9,11,12,13,14]. Studies have shown that ascites contribute to tumor heterogeneity, modulate the expression of integrins, and alter the activity of matrix-degrading enzymes in ovarian cancer [5,11,15,16,17]. These effects by ascites on ovarian tumors have largely been studied in the context of the soluble factors (e.g., cytokines and metabolites) and cellular components (e.g., tumor cells and stromal cells) that are commonly found in ascites. In addition to providing a cell- and protein-rich environment for cancer growth and progression, ascitic fluid also confers physical stress, such as flow-induced shear stress, on tumors [18,19,20,21,22,23,24,25,26]. Natural currents of ascitic fluid result, in part, from organ mobility, changes in negative subdiaphragmatic pressure, gravity, and recesses formed by anatomical structures [4,27,28,29,30]. An area that remains understudied is the effect of fluid shear stress on response to chemotherapy and the modulation of molecular pathways associated with aggressive disease [11,16,17,31].

Fluid stress has been shown to influence tumor migration and invasion and to induce changes in the morphologic, genetic, and protein expression profiles of cancers, including ovarian cancer [18,19,20,21,22,23,24,25,26]. We have previously shown a post-translational upregulation and activation of the epidermal growth factor receptor (EGFR) in a 3D perfusion model for ovarian cancer [25]. The EGFR is a transmembrane receptor tyrosine kinase that regulates proliferation, growth, and survival [32,33,34]. High EGFR expression in ovarian cancer is associated with increased proliferation and invasiveness [35,36,37,38] and may be a negative prognostic indicator of survival [39,40,41,42]. Flow-driven EGFR upregulation is likely a result of decreased receptor degradation and increased recycling [25]. A concomitant, transcriptionally-regulated, flow-induced decrease in E-cadherin, along with an increase in vimentin expression, was observed [25]. The impact of flow-induced shear stress on EGFR-associated survival pathways and resistance to platinum therapy remains understudied.

Primary treatment of advanced-stage ovarian cancer typically involves debulking surgery followed by platinum and taxane-based chemotherapy or in some cases, hormonal therapy [43]. Intraperitoneal treatments provide the opportunity to realize a multi-fold increase in local drug concentration compared to intravenous administration [43]. In patients with stage III ovarian cancer who underwent an optimal debulking surgery, administration of intraperitoneal platinum-taxane combination chemotherapy resulted in a median overall survival of 65.6 months, compared to 49.7 months with intravenous therapy [43]. Hyperthermic intraperitoneal chemotherapy (HIPEC) at the time of cytoreductive surgery for peritoneal carcinomatosis has also produced encouraging results, particularly for ovarian cancer and mucinous tumors of the gastrointestinal tract [44,45,46]. In a Phase III trial for patients with stage III epithelial ovarian cancer, surgery with administration of HIPEC with cisplatin (100 mg/m^2^) improved the median overall survival to 45.7 months, compared to 33.9 months in the surgery group [45].

Photodynamic therapy (PDT) is a light-based treatment modality that is mechanistically distinct from conventional agents and has been studied in the clinic for disseminated intraperitoneal tumors [47,48]. PDT reverses chemoresistance, synergizes with chemotherapeutics and biologics, and overcomes compensatory survival pathways used by cancers to evade treatment [48,49,50,51,52]. PDT uses near-infrared (NIR) light to excite a photoactivatable molecule called a photosensitizer [48]. The activated photosensitizer produces highly reactive molecular species (RMS, e.g., ^1^O_2_, •OH) that confer toxicity to nearby targets. Cytotoxicity from PDT is governed by the localization of the photosensitizer, spatial confinement of light, and the short distances over which the RMS remain active [48]. Above a critical threshold dose, the RMS-induced damage leads to cell death, while the remaining cells are primed for subsequent treatment with a chemo or biological agent. This priming results from modulating the balance of pro- and anti-apoptotic regulatory proteins and (depending on the photosensitizer) direct damage to subcellular organelles.

Photoimmunotherapy (PIT) is a variant of PDT involving the targeted delivery of the photosensitizer via an antibody conjugate to enhance tumor selectivity in complex sites such as the peritoneal cavity [50,53,54,55,56,57,58]. Previous studies [59,60] have shown that PIT with an EGFR-targeted photoimmunoconjugate (PIC) minimizes damage to surrounding healthy tissue [59,61]. In the clinic, harmless NIR light can be spatially confined and delivered through fiber optics and balloons to the peritoneum and areas of resection to activate photosensitizers [62,63,64,65], thus giving PIT the advantage of selectivity to the tumor-invaded peritoneum. Building on these and other efforts, we investigate here, for the first time, the influence of flow-induced shear stress on carboplatin resistance and the activity of the EGFR, mitogen-activated protein kinase/extracellular signal-regulated kinase (MEK), extracellular signal-regulated kinase (ERK) pathway in a 3D perfusion model for ovarian cancer. In addition, a low-dose PIT approach is leveraged to target EGFR overexpression in tumors grown under flow-induced shear stress.

## 2. Experimental Section

### 2.1. Cells and Cell Culture

Human epithelial ovarian cancer cell line, NIH:OVCAR-5 (OVCAR-5), was a kind gift from Thomas Hamilton at Fox Chase Cancer Institute (Philadelphia, PA). Cells were grown in 2-dimensional (2D) monolayer in a T75 flask in RPMI 1640 (#10-040-CV, Cellgro, Corning, New York, NY, USA) with 10% heat-inactivated fetal bovine serum (FBS) in the presence of 100 U/mL penicillin and 100 µg/mL streptomycin at 37 °C and 5% CO_2_ in a humidified incubator. The cells were subcultured every 3 to 4 days depending on cell density. A new stock of OVCAR-5 cells was used once the passage number exceeded 30. Cells were tested regularly for *Mycoplasma* infection using the MycoAlert kit (#LT07-710, Lonza, Basel, Switzerland).

### 2.2. Synthesis and Characterization of Photoimmunoconjugates (PICs)

The PICs were prepared by conjugating the photosensitizer, benzoporphyrin derivative (BPD), to an anti-EGFR monoclonal antibody, cetuximab (Erbitux, Bristol-Meyers Squibb, New York, NY, USA), using carbodiimide crosslinker chemistry following the established protocols [50,57,58,66,67]. PEGylation of cetuximab before BPD conjugation was necessary to prevent the aggregation of cetuximab-BPD conjugates. Briefly, 0.2 mL of mPEG-NHS (10,000 MW; 4 mg/mL) in dimethyl sulfoxide (DMSO) was added dropwise to 2 mL of cetuximab (2 mg/mL) and allowed to stir overnight at 400 rpm at room temperature. PEGylated cetuximab was reacted with the N-hydroxysuccinimidyl ester of BPD (BPD-NHS) at a 1:9 molar ratio for 4 h. The resulting PICs were purified using a 7kDa MWCO Zeba spin desalting column pre-equilibrated with 30% DMSO in PBS and then washed in a 20 mL 30 kDa MWCO centrifugal filter tube (Amicon, MilliporeSigma, Burlington, MA, USA) three times with 5% DMSO in PBS prior to storage at 4 °C. Sodium dodecyl sulfate-polyacrylamide gel electrophoresis (SDS-PAGE) was used to identify and monitor the purity of cetuximab-BPD, which is typically less than 1% free BPD. The BPD concentration of PIC was estimated by absorbance in DMSO using the established molar extinction coefficient of BPD in DMSO (~34,895 M^-1^cm^-1^ at 687 nm). The protein content of PIC was evaluated by BCA protein assay (#23225, Pierce, ThermoFisher Scientific, Waltham, MA, USA). BPD conjugation efficacy was determined by dividing the amount of antibody-conjugated BPD in the purified PIC by the total amount of BPD added initially. We have previously determined the conjugation efficiency to be approximately 66% (i.e., 6-7 BPD molecules per cetuximab) and this reaction does not impair the selective binding and internalization of cetuximab in EGFR-positive cancer cells.

### 2.3. Static 3D Culture

Growth Factor Reduced (GFR) Matrigel (Corning, cat. #354230, lot #5173009, 7016289) was thawed overnight on ice. To form the Matrigel bed, 250 µL/well Matrigel was transferred to a pre-chilled 24-well plate. For imaging experiments, 24-well black-wall glass-bottom plates (#662892, Sensoplate, Greiner Bio-One, Monroe, NC, USA) were used, and for other static experiments, standard 24-well plates (#353047, Corning, New York, NY, USA) were used. The Matrigel-coated plates were then incubated at 37 °C for 20–30 min to allow for Matrigel polymerization. OVCAR-5 cells grown in monolayer were washed in phosphate-buffered saline (PBS), trypsinized, and resuspended in complete culture medium. Next, 10,000 OVCAR-5 cells in 1 mL 2% Matrigel-containing complete culture medium were added to each well and incubated for seven days. No treatment controls were handled identically, but they did not receive carboplatin or PIC in the medium.

### 2.4. Carboplatin Treatments

Culture medium was changed every 3 to 4 days. On day 7, 1 mL of complete culture medium with a range of different carboplatin doses in triplicate (0, 10, 25, 50, 100, 250, and 500 µM) (#S1215, Selleck Chemicals, Houston, TX, USA) was added to each well and incubated for an additional four days.

### 2.5. PIT Treatments

Culture medium was changed every 3 to 4 days. On day 6, 1 mL of complete culture medium containing 1µM BPD-equivalent to the PIC was added to each well and incubated for 24 h, based on extensive studies demonstrating the kinetics of PIC processing and delivery of photochemically available photosensitizers [49,59,60,61,67,68,69,70]. The following day, PIC-containing medium was removed from each well and replaced with fresh complete culture medium. For PIT, wells were exposed to a range of energy densities in triplicate (0, 10, 15, 20, 25, 40, 50 J/cm^2^ at an irradiance of 50 mW/cm^2^) using a 690 nm laser (Model 7404, Intense Inc., North Brunswick, NJ, USA). Plates were then placed back in the incubator for an additional four days.

### 2.6. Chip Assembly and 3D Flow Culture

Chips were prepared as described previously [25,71,72,73] with the addition of braces to prevent leakage (Figure S1). Briefly, 3.175 mm thick polymethyl methacrylate (PMMA)(McMaster Carr, Elmhurst, IL, USA) was used for the top of the chip and for the braces (Figure S1A). Medical-grade double-sided adhesive (DSA) film (white, 254 μm thick, 10 mil, ARcare #92660, Adhesives Research, Glen Rock, PA, USA) was used to provide the channel height. Three channels were cut in to the DSA (VersaLASER, Universal Laser Systems Inc., Scottsdale, AZ, USA), each with a width of 4 mm and 3 mm between the channels. The channel inlet and outlet regions were fabricated with a 127° angle to facilitate fluid entrance to and exit from the channels. The inlet and outlet ports of microchannels were 2.2 mm in diameter and were positioned 5 mm from the edge of the chip. The channel length was defined as the distance between the inlet and outlet. All components were sterilized using 100% ethanol followed by ultraviolet irradiation for 1 h inside a cell culture hood. Chip assembly was performed in a laminar flow hood. Glass coverslips (Corning, 0.13-0.17mm) with DSA films attached were chilled on an ice pack. Growth Factor Reduced Matrigel (#354230, BD Biosciences, San Jose, CA, USA) was spread evenly within each channel (20 μl/channel). A PMMA layer with holes cut for tubing (VersaLASER, Universal Laser Systems Inc., Scottsdale, AZ, USA) was used to cover the DSA film, forming Matrigel-coated perfusion channels. The chip was then placed between two PMMA braces (Figure S1B) to prevent leaks during extended culture and treatment. The chips were incubated at room temperature, allowing gelation of Matrigel within the channels. Next, gas-permeable silicon tubing (#3350 TYGON silicone, Cole-Parmer, Vernon Hills, IL, USA) was attached to the inlet and outlet ports (Figure S1C). Epoxy glue (5 Minute Epoxy, Devcon, Hartford, CT, USA) was used to seal the external tubing edges at the inlet and outlet. For flow experiments, 0.5x10^6^ cells in 500 µL of culture medium were initially pumped into each inlet tube at a rate of 100 µL/min for 5 min. Informed by previous protocols [25,71,72,73], a physiologically relevant flow rate of 2 µL/min (3 dyne/cm^2^) was maintained during 3D culture with 2% Matrigel-containing complete medium for seven days and for subsequent treatment with carboplatin or PIT. No treatment controls were handled identically, but they did not receive carboplatin or PIC in the medium.

### 2.7. Carboplatin Treatments

On day 7, carboplatin (500 µM), in complete culture medium, was pumped through the channels for an additional four days at 2 µL/min.

### 2.8. PIT Treatments

On day 6, 1 µM-BPD equivalent of PIC in complete culture medium was pumped through the channels at 2 µL/min for 24 h, based on extensive studies demonstrating the kinetics of PIC processing and delivery of photochemically available photosensitizers [49,59,60,61,67,68,69,70]. On day 7, the syringes were replaced with complete culture medium and the medium was pumped for an additional 4 h before PIT application to ensure that the PIC-containing medium inside the channels was replaced with fresh culture medium. While connected to the tubing and pump, all three channels on the chip that received PIC were subsequently treated with 690 nm light using an energy density of 15 J/cm^2^ at an irradiance of 50 mW/cm^2^ (Model 7404, Intense Inc., North Brunswick, NJ, USA).

### 2.9. Simulation of Fluid Dynamics

**Figure 2:**
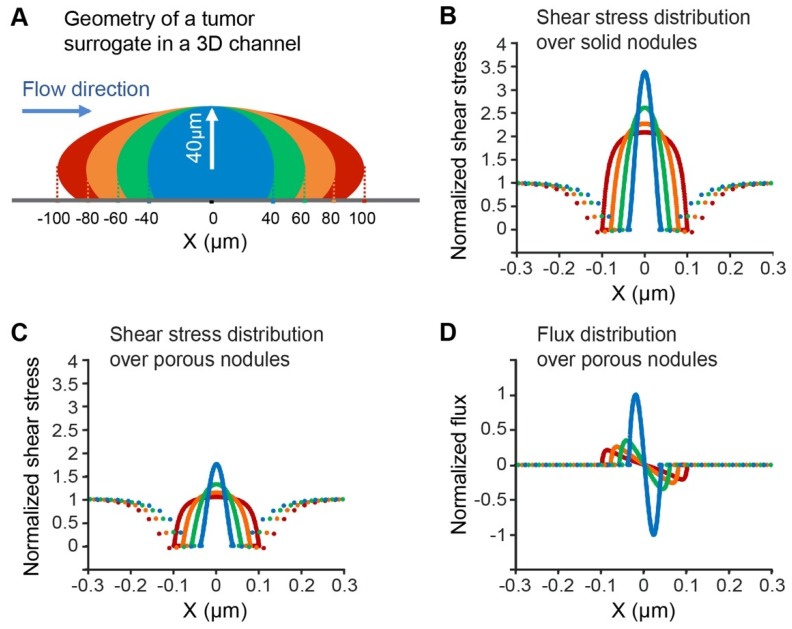
(**A**) Geometry of the micronodule located at the center of the microchannel. The flow velocity is in the X-direction. The nodule is modeled as an ellipse with a semi-minor axis of 40 μm in the Z-direction. The semi-major axis varies from 40-100 μm in the X-direction. The section over which the fluid dynamics are studied is the middle part of the channel with dimensions 4 mm along the Y-axis and 250 μm along the Z-axis. The nodule is located at (0, 20 μm). The black dotted line shows the centerline of the largest nodule. (**B**) Shear stress distribution over the surface of the solid micro-nodule on the XZ-plane. (**C**) Shear stress distribution over the surface of the porous micro-nodule on the XZ-plane. (**D**) Flow flux distribution over the centerline of the porous micro-nodule on the XZ-plane. The flux enters the surface at the left and leaves at the right.

To characterize the dynamics of the flow and the hydrodynamic forces applied on the surface of cancer nodules, a mathematical model is solved over a domain of the 2D micro-channel. Since the flow rate is very small, the flow velocity profile in the center of the microchannel is not affected by the velocity profile close to the inlet and outlet. For this reason, the fluid mathematical model is solved over the central part of the channel. Furthermore, to understand the mutual interaction between the flow dynamics and the nodule geometry, the equations are solved for a single nodule attached to the center of the channel. The dimension of the tumor is chosen based on the reported images of the tumor cultured under flow in [25]. The tumor surrogate has an ellipsoid shape with semi-minor axes of 40 µm in Z-direction. The semi-major axes varied from 40-100 µm in X-direction. The protrusion of the tumor is 60 µm (Figure 2A).

Modeling the flow dynamics around the nodule is presented in two steps. In the first step, it is assumed that a nodule is an impermeable solid object with no porosity or flexibility; this step is particularly important to study the effect of the environment geometry on the hydro-dynamical forces. The fluid dynamics in the microchannel and around the nodule is represented by Stokes equations; the fluid flow and pressure profiles are defined as the solutions to these equations [74]. The effect of external forces can be ignored in these studies. The velocity at the walls of the microchannel, as well as at the surface of the nodule, is set by the no-slip boundary condition. The inlet velocity is assumed to be 0.033 mm/s and outlet pressure is the atmospheric pressure.

To have a more realistic model, the nodule is assumed to be permeable as it is in real-life. In addition to the Stokes equations, which define the flow dynamics around the nodule, the Brinkman equations are solved for the flow inside the nodule.

The modeling parameters are adapted from published articles [75,76,77] based on the tumor properties under flow in the perfusion channel as reported previously [25]. The mathematical equations representing the flow dynamics are solved using COMSOL Multiphysics 5.4 (COMSOL, Inc, Burlington, MA, USA), based on the finite element method (FEM). The shear stress on the nodule surface is obtained from the flow velocity profile.

### 2.10. Western Blot and Antibodies

For harvesting cells from the Matrigel bed of static cultures, the medium was removed and the cells were incubated with pre-chilled Cell Recovery Solution (Corning, 354253) according to the manufacturer’s protocol at 4°C for two h on a horizontal shaker at 100 rpm. The cell suspension was collected, pelleted, and washed twice with cold PBS containing a phosphatase and protease inhibitor cocktail (#78440, ThermoFisher Scientific, Waltham, MA, USA), which was then aspirated to obtain only the tumor pellet. The cell pellet was lysed in modified RIPA buffer in the presence of the phosphatase and protease inhibitors as described previously [78]. Protein concentration was estimated using BCA assay (#23225, Pierce, ThermoFisher Scientific, Waltham, MA, USA). For running SDS-PAGE, 20 µg of protein/lane was loaded on to a 4–20% precast gradient gel (#4561093, Bio-Rad Laboratories, Hercules, CA, USA). Western Blots were performed as previously described [79]. The following antibodies were used for immunoblots: MEK (#4694, Cell Signaling Technology (CST), Danvers, MA, USA), ERK1/2 (CST, 8201), phospho-ERK1/2 (CST, 8201), Vinculin (CST, 13901), paxillin (CST, 12065), phospho-paxillin (CST, 2541), FAK (CST, 3285), phospho-FAK (CST, 8556), EGFR (#sc-03 (1005), Santa Cruz Biotechnology, Dallas, TX, USA), and GAPDH (#ab8245, Abcam, Cambridge, MA, USA). All western blots represent three independent experiments with pooled samples from three wells (static) or 2 to 3 lanes (flow) per experiment. Each of the independent experiments were blotted three times to verify reproducibility.

### 2.11. Confocal Imaging of 3D Tumors

On day 11, a live cell stain of 4 µM calcein AM (#C3100MP, Invitrogen, ThermoFisher Scientific, Waltham, MA, USA) in PBS was prepared. In the case of static plate imaging, the medium was removed and the cells were incubated with the staining solution for 30 min. For thresholding purposes and to determine the dynamic range for calcein signal, cells were either treated with 2% formalin or left untreated as previously described [80,81,82,83]. The plate was mounted on a laser scanning confocal microscope (FV1000, Olympus, Tokyo, Japan) and a single z-plane at the center of each well was obtained by taking 2-by-2 mosaic images (512 × 512 pixel each) with a 4X objective lens (NA 0.16). For calcein fluorescence imaging, an excitation wavelength of 488 nm was used, and emission was collected from 510 to 520 nm.

For the flow experiments, 900 µL of the staining solution was pumped at a rate of 10 µL/min for 90 min in an incubator through each channel. The inlet and outlet tubes were clamped and cut, the bottom brace was removed, and the chip was mounted on a custom-made 3D printed microscope stage-adapter for confocal imaging (Figure S1D). The full channel was imaged by taking multiple mosaic images (512 × 512 pixel) with a 10× (N.A. 0.4) objective lens in multiple Z-planes (~15–20 planes per channel, step size: 10 μm) using the confocal microscope. The same excitation and emission parameters were used for imaging. The images were stitched together and analyzed using custom algorithms (qVISTA) [83,84], as described in the next section.

### 2.12. Image Analysis and Statistical Evaluation for Normalized Live Tumor Area

To quantify response to treatment in 3D cell cultures grown under flow or static media conditions, sets of confocal image data were processed using a custom set of image analysis routines that were developed in MATLAB (Mathworks, Natick, MA, USA), based on previously reported methods [25,81,83,84,85]. For both flow and non-flow culture groups, the area of viable disease was obtained via segmentation of fluorescence from calcein-positive regions as described previously. In flow channels, the regions of live tumor were found to occupy multiple focal planes, which were captured as multi-area Z-stack mosaics. These image sets were stitched laterally and projected (maximum intensity projection, MIP) in the Z-dimension to capture growth throughout the full depth of the channel prior to image segmentation. In non-flow cultures, cells uniformly remained on the surface of the Matrigel bed, such that all nodules were captured in a single focal plane without the need for obtaining a Z-stack or projecting intensity across planes. Otherwise, segmentation and subsequent analysis were performed identically across the flow and non-flow groups. In practice, image mosaics (13 × 3 tiles) were sufficiently large (~700 megabytes) that additional adaptations were required for memory management while processing. Briefly, image data for individual tiles were cleared from memory after segmentation of calcein-positive objects in the tile and the immediately bordering tiles (as needed until the full extent of an object was reached) so that features spanning across adjacent tiles would be recorded without breaking up into multiple spurious individual features. In this manner, total disease area was segmented, summed over each channel, and normalized to its internal respective control group within each growth condition.

### 2.13. Carboplatin (Platinum) Uptake

Cells grown on Matrigel-overlay were harvested using Cell Recovery Solution as mentioned earlier. Cells were washed twice in cold PBS, pelleted, and stored in −80 °C until use. A fraction of the cell pellet was used for protein estimation (#23225, Pierce, ThermoFisher Scientific, Waltham, MA, USA) after lysing the cells using RIPA buffer. The remaining cell pellet was used for ICP-MS. The cells were pipetted into Teflon vials and their mass was recorded. Then 1 mL of concentrated (67–70%) trace metal grade nitric acid (#87003-261, VWR International, Avantor, Radnor, PA, USA) was added to each vial. After the addition of nitric acid, 100 µL of concentrated (30% to 32%) trace metal grade hydrogen peroxide (#87003-224, VWR, VWR International, Avantor, Radnor, PA, USA) was added. The vials were placed on a hotplate (150–175 °C) and left for digestion for 8 h. Each sample was transferred to a 15 mL centrifuge tube and filled with Nanopure water to make up the volume to 15 mL. The final weight was recorded. This final solution was used to quantify platinum concentration by inductively coupled plasma mass spectrometry (ICP-MS). The amount of platinum present in each sample was normalized to the protein content of the respective sample.

### 2.14. Statistical Analysis

Mean normalized tumor areas in the flow cultures were compared to mean normalized tumor areas in the static cultures for the respective round. Statistical analyses were performed using Prism 8.0 software (GraphPad, San Diego, CA, USA): * *p* ≤ 0.05, ** *p* ≤ 0.01, *** *p* ≤ 0.001.

## 3. Results

### 3.1. Perfusion 3D Tumor Model For Ovarian Cancer

The perfusion model previously used to assess the effect of flow-induced shear stress on the genetic, molecular, and morphologic features of ovarian cancer in 3D culture over 7 days [25] was modified to evaluate response to carboplatin treatment and PIT in the present study. Braces to prevent leaks and reservoirs to hold the tubing during prolonged culture and carboplatin treatment were added to the second-generation perfusion chamber, which enabled the treatment response studies reported here (Figure S1A–C and movie S1). In the modified perfusion chambers, cultures of adherent NIH: OVCAR-5 cells were established on stromal beds of Matrigel and formed 3D tumors under flow (Figure 1B) that were morphologically distinct from corresponding static 3D cultures (Figure 1C) consistent with previous findings [25]. Subsequent treatment with 500 µM carboplatin resulted in a noticeable decrease in tumor viability in static cultures (Figure 1C), in contrast to flow cultures (Figure 1B), as observed by calcein fluorescence (green). A custom-made 3D-printed chip-holder to mount and image the chip on the microscope stage enabled, for the first time, full chip imaging in the X, Y, and Z-planes for all channels (Figure S1D).

### 3.2. In Silico Simulation of Shear Stress on Tumor Nodules

Computational modeling was used to evaluate fluid dynamics in the perfusion chamber and to estimate the forces experienced by the nodules. The fluid flow in the chamber is described by the steady-state Navier-Stokes equations with the assumption of incompressible flow and the flow inside the permeable nodule is described by the Brinkman equations. The shear stress, considered here, is defined by the variation of the tangential velocity component normal to the nodule’s surface; the derived shear stress values are normalized with respect to the wall shear stress, which is 0.008 dyn/cm^2^ and was calculated by solving the velocity field described by the Navier-Stokes equations. A schema of flow-induced shear stress for a solid non-porous nodule of four different sizes is depicted in Figure 2A. As shown in Figure 2B, increasing the radius of impermeable nodules in the X-axis (i.e., in the direction of flow) leads to a reduction in the maximum shear stress experienced by the solid nodule, while the shear stress profile remains symmetric relative to the top of the nodule. These phenomena can be explained by the fact that elongating the nodule in the flow direction reduces the maximum variations in the flow velocity profile and thus, the maximum shear stress. Adding porosity and permeability to the structure of the nodule does not change the overall trend, however the magnitude of the shear stress experienced by the nodule is noticeably smaller as the flow can permeate the tumor structure (Figure 2C). As shown in Figure 2D, the pattern of the flow flux penetrating the nodule varies in relation to tumor geometry. Here, the positive magnitude of the flux indicates that the flow is entering the nodule and the negative sign indicates the flow exiting the nodule. For the sphere-shaped nodule, the flow tends to enter on the left hemisphere and leave from the right hemisphere symmetrically; for the elongated nodule, the same occurs over a longer distance. This flow pattern reflects the distribution of the shear stress (Figure 2C); that is, the highest shear stress occurs where the flow runs parallel to the nodule surface, hence zero flux into the nodule. The maximum flux into the nodule occurs where the normal component of the velocity is the largest: 1.06 nm/s for the spherical nodule. This velocity is the scaling factor in Figure 2D. In summary, our numerical results indicate that porosity is an important factor that must be considered in deriving the hydrodynamic forces. The simulations also predict that the tumor nodules experience a wide range of shear stress depending on their geometry and size. In fact, the previous study reported that the nodules grown in microchannel exhibited diversity in their geometry and size [25]; thus, the maximum shear stress differs from point to point, both in the channel and in the nodule.

### 3.3. Carboplatin Dose-Response and Platinum Uptake in Static 3D Ovarian Cancer Cultures

**Figure 3:**
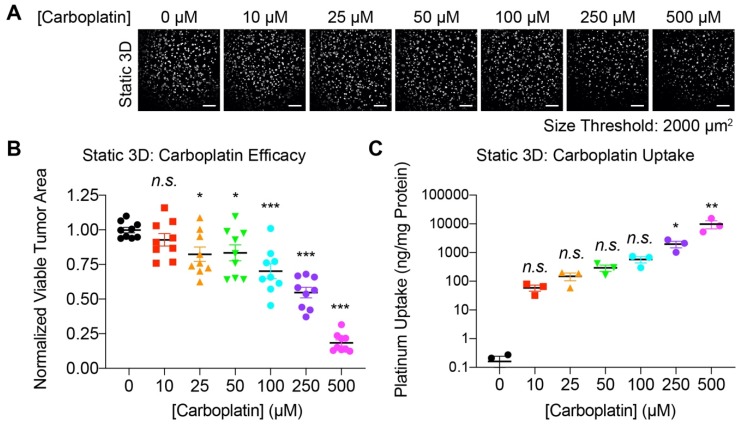
Cytotoxic response in carboplatin-treated 3D OVCAR-5 cultures under static conditions. (**A**) Representative confocal images of 3D tumors treated with carboplatin (0-500 μM) for 96 h showing a dose-dependent reduction in viable tumor (calcein signal). (**B**) Image-based quantification of normalized viable tumor area in 3D OVCAR-5 cultures following treatment with increasing doses of carboplatin. A minimum nodule size cut-off of 2000 µm^2^ (clusters of ~15–20 cells) was applied to the fluorescence images for quantitative analysis of the normalized viable tumor area. (One-way ANOVA with Dunnett’s post hoc test; n.s., not significant; * *p* < 0.05; *** *p* < 0.001; N = 9) (**C**) Inductively coupled plasma mass spectrometry (ICP-MS)-based quantification of carboplatin uptake in static 3D OVCAR-5 tumors shows a dose-dependent increase in platinum levels, up to 9774 ± 3,052 ng/mg protein at an incubation concentration of 500 μM carboplatin. (One-way ANOVA with Dunn’s multiple comparisons test; n.s., not significant; * *p* < 0.05; ** *p* < 0.01; N = 3). Results are expressed as mean ± standard error of mean (SEM). Scale bars: 500 μm.

OVCAR-5 cells were grown in 3D static and flow cultures with complete growth medium for seven days, at which point they formed spherical tumor nodules (static) or elongated continuous tumors (flow) (Figure 1). Next, the cells in static culture were treated with different doses of carboplatin (0, 10, 25, 50, 100, 250, 500 µM) for an additional four days. On day 11, cells were stained with calcein-AM and live tumor area was determined by in situ confocal microscopy. For quantitative analysis of the resulting fluorescence images (Figure 3A), a minimum nodule size cut-off of 2000 µm^2^ (clusters of ~15–20 cells) was used to establish the normalized tumor area as the metric to evaluate the treatment outcomes [25,80,81,83,86].

Figure 3B shows live tumor area following treatment with a range of carboplatin doses in static 3D cultures of ovarian cancer. The data represent normalized tumor area from an individual well (solid shapes), mean normalized tumor area (solid line) for three independent experiments in triplicate, and standard error of the mean (error bar). Treatment with 10 µM carboplatin resulted in non-significant changes in mean normalized tumor area (0.93 ± 0.04). Following treatment with 25 and 50 µM carboplatin, a modest and comparable, but significant, reduction in mean normalized tumor area was observed (0.82 ± 0.05 and 0.83 ± 0.06, respectively, *p* < 0.05). At a dose of 250 µM, the mean normalized tumor area was reduced to nearly half of the no treatment controls (0.55 ± 0.04, *p* < 0.05). A further increase in the dose to 500 µM carboplatin resulted in a > 80% tumoricidal effect (mean normalized tumor area: 0.18 ± 0.02, *p* < 0.05). Size distributions of adherent 3D ovarian tumors following treatment with 250 and 500 µM carboplatin are shown in Figure S2. Carboplatin causes cell death by irreversibly binding to DNA and inducing the formation of DNA adducts [87,88,89,90]. Accumulation of these adducts depends on the intracellular concentration of carboplatin. Therefore, intracellular concentration of carboplatin was quantified by ICP-MS (Figure 3C) for each of the doses used in the cytotoxicity studies (Figure 3B). A dose-dependent increase in carboplatin uptake was observed in the static cultures, ranging from 60.4 ± 14.08 ng/mg protein at 10 µM carboplatin (mean ± sem, n.s., *p* > 0.05) to 9774 ± 3052 ng/mg protein at 500 µM carboplatin (mean ± sem, *p* < 0.05).

### 3.4. Carboplatin Response and Platinum Uptake in 3D Ovarian Cancer Cultures Under Flow

**Figure 4:**
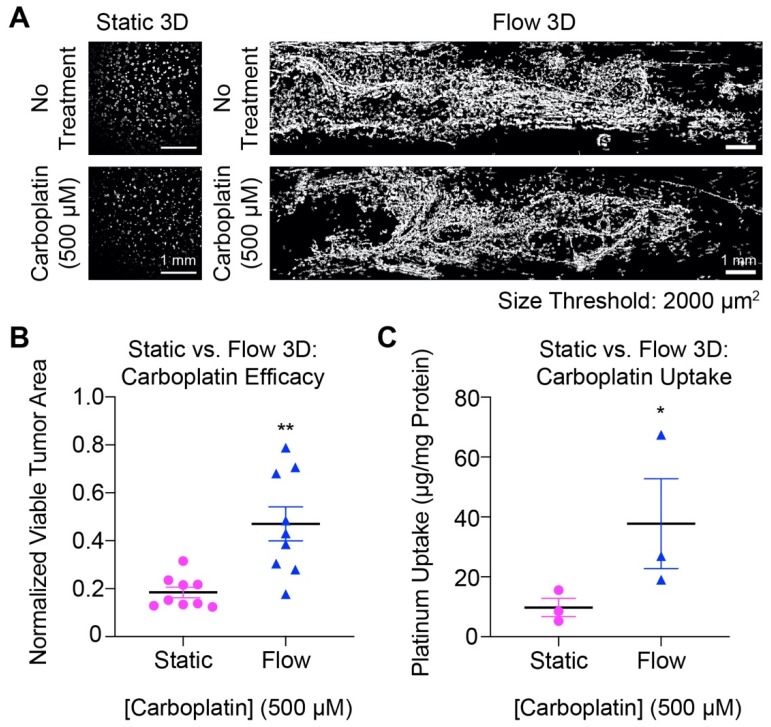
Comparison of the cytotoxic response in carboplatin-treated 3D OVCAR-5 tumors under static and flow conditions. (**A**) Representative confocal images of viable tumor (calcein signal) treated for 96 h with 500 μM carboplatin under static (Left) and flow conditions (Right), and respective no treatment controls. (**B**) Tumors treated with 500 μM carboplatin under flow show 2.6-fold higher normalized viable tumor area, compared to tumors treated with 500 μM carboplatin under static conditions. (Two-tailed t test; ** *p* < 0.01; N = 9). **(C)** The intracellular concentration of platinum was 3.9-fold higher in 3D tumors grown under flow than comparable static 3D cultures (Mann-Whitney test; * *p* ≤ 0.05; N = 3). A minimum nodule size cut-off of 2000 µm^2^ (clusters of ~15–20 cells) was used to quantify the normalized viable tumor area. Results are expressed as mean ± standard error of mean (SEM). Scale bars: 1 mm.

Using a minimum nodule size cut-off of 2000 µm^2^, Figure 4A,B show that OVCAR-5 tumors grown under flow-induced shear stress were less responsive to treatment with 500 µM carboplatin than comparable tumors in static cultures. The mean normalized area of tumors grown under flow-induced shear stress and treated with 500 µM carboplatin was significantly higher (0.47 ± 0.07) compared to static cultures (0.18 ± 0.02) (*p* < 0.05) (Figure 4B). To determine whether this reduction in carboplatin efficacy in tumors grown under flow-induced shear stress was a result of poor chemotherapy delivery, the intracellular concentration of platinum was quantified by ICP-MS (Figure 4C). Platinum concentration in tumors grown under flow-induced shear stress was significantly higher (37,788 ± 15,007 ng/mg protein) than static cultures (9773 ± 3,052 ng/mg protein) (*p* < 0.05).

Image analysis using a range of size cut-offs. To define a live tumor from the background, we evaluated minimum nodule size cut-offs ranging from 500 µm^2^ (clusters of <10 cells) up to a maximum of 3000 µm^2^ (clusters of ~25–30 cells) using the “adaptive thresholding” function of MATLAB 2017b (Figure S3 A–E and Figure S4). The dose-dependent decrease in viable tumor area for each carboplatin-treated group in static culture was non-significantly different across all threshold size cut-offs evaluated (Figure S3F, *p* > 0.05, ANOVA).

In the perfusion chambers (Figure S5), individual images (512 × 512 pixels) were taken and stitched together to form a 13 × 3 tile mosaic with 10% overlap to cover the entire channel. Mosaic images of calcein fluorescence were stitched together for each Z-plane first and then projected into a single plane to obtain a “maximum intensity projection” (MIP). A binarized mask for each Z-plane was created based on the same segmentation algorithm used for static cultures and projected to form a composite binarized mask which was then multiplied by the corresponding MIP, as shown schematically in Figure S5A. Representative fluorescence image mosaics and corresponding binarized masks for each z-plane are shown in Figure S5B,C, respectively. The impact of varying minimum nodule size cut-offs on the composite binarized masks and binarized mask-MIP product images for the same range of minimum nodule size cut-offs is shown in Figure S5D,E [84]. Finally, live tumor area was determined by multiplying the MIP and “composite binarized mask” and thresholding for minimum nodule size. For each threshold size, the live tumor area was internally normalized to its own “no treatment” control group and presented in Figure S6A–F. There was no significant difference in live tumor area for size thresholds ranging from 500 to 3000 µm^2^ based on a multivariate ANOVA (*p* > 0.05) (Figure S6G–H). Analysis of treatment response for each of the other size cut-offs is provided in (Figure S7), showing the same trends in carboplatin efficacy between flow and static cultures.

### 3.5. Molecular Changes Associated With Flow-Induced Shear Stress

**Figure 5:**
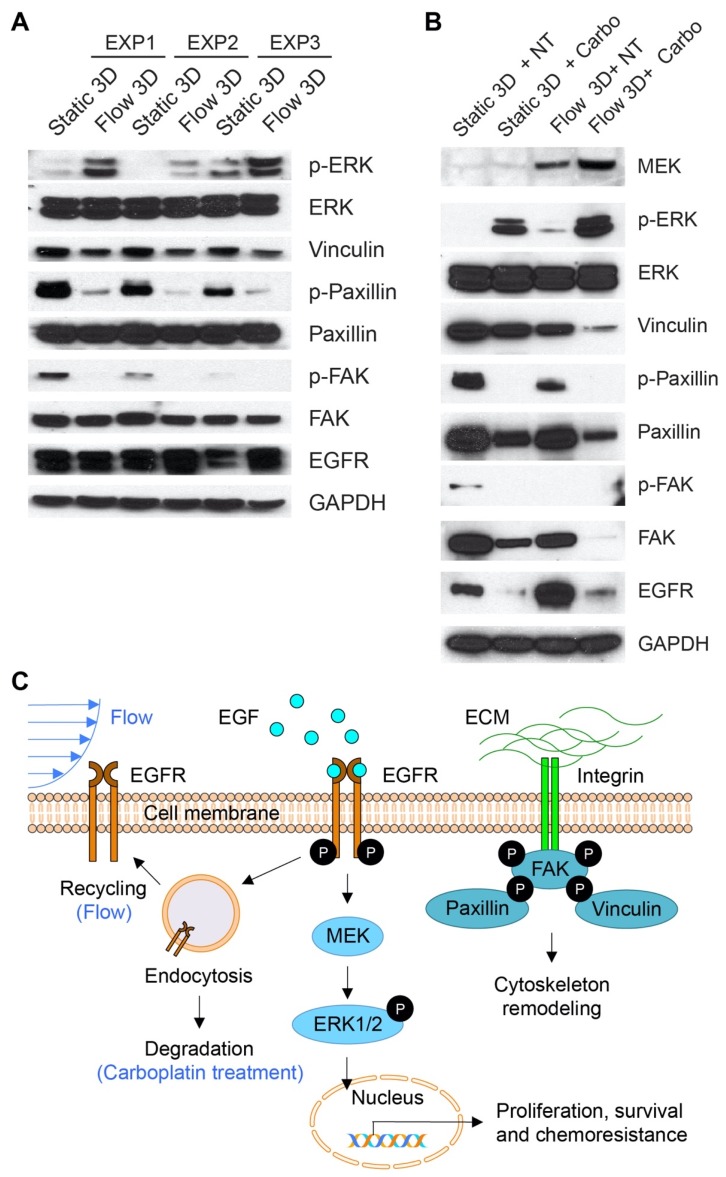
The effects of flow-induced shear stress on 3D ovarian cancer biology. (**A**) Western blot analysis of OVCAR-5 tumors was performed 7 days after culture under static or flow conditions. A flow-induced increase in EGFR and p-ERK, compared to static cultures, was observed. Conversely, a reduction in p-FAK, p-Paxillin, and Vinculin was observed under flow, relative to static conditions. (**B**) Western blot analysis of 3D OVCAR-5 tumors was performed 11 days after culture under static or flow conditions, including 4 days of treatment with 500 µM carboplatin, and respective controls. In both static and flow 3D cultures, carboplatin treatment resulted in downregulation of EGFR, FAK, p-Paxillin, Paxillin, and Vinculin. Upregulation of p-ERK was observed after carboplatin treatment in both static and flow 3D cultures. (**C**) Baseline levels of EGFR activity and expression are maintained by a complex array of factors, including recycling and degradation of the activated receptor complex. Flow-induced shear stress has been shown to cause a posttranslational up-regulation of EGFR expression and activation, likely resulting from increased receptor recycling and decreased EGFR degradation. Activation of EGFR results in ERK phosphorylation to induce gene expression, ultimately leading to cell proliferation, survival, and chemoresistance. FAK and other tyrosine kinases are activated by the engagement of integrins with the ECM. Subsequent phosphorylation of paxillin by FAK not only influences the remodeling of the actin cytoskeleton, but also modulates vinculin activation to regulate mitogen-activated protein kinase (MAPK) cascades, thereby stimulating pro-survival gene expression.

We previously reported that flow-induced shear stress leads to overexpression of EGFR [25], a poor prognostic factor. Here, 3D nodules were collected from both static and flow cultures (no treatment and carboplatin treated) and protein expression was estimated by western blot (Figure 5). Expression of phosphorylated (phospho)-ERK1/2 significantly increased in cells grown under flow compared to static cultures (Figure 5A). However, total ERK1/2 levels remained unchanged. Consistent with our previous findings, EGFR expression was also elevated in cells under flow compared to static cultures.

Next, we investigated whether the expression of focal adhesion proteins that have been implicated in cancer progression and metastasis [91,92] was altered in 3D tumors grown flow-induced shear stress (Figure 5A). It was found that total expression of vinculin, paxillin, and FAK remained relatively unchanged in cells under flow compared to the static culture. However, expression of phospho-paxillin and phospho-FAK were significantly decreased in cells grown under flow compared to static cultures.

The EGFR/ERK signaling pathway is upregulated in many cancer types including ovarian cancer and is frequently implicated in treatment resistance [39,40,41,42,93]. It was previously shown that flow-induced mechanical stress mediates a post-translational increase in the expression of EGFR in ovarian cancer cells. Phosphorylation of EGFR at Y1173 was also increased in ovarian cancer cells under flow compared to static culture [25]. Here, the role of this pathway in the carboplatin resistance, shown in Figure 4, was investigated in tumors grown under flow and static conditions and treated with carboplatin (Figure 5B). It was found that the expression of phospho-ERK1/2 (at Thr202/Tyr204) was dramatically increased in OVCAR-5 cells grown under flow compared to static cultures in all the experimental replicates, whereas the total ERK expression remained the same. Interestingly, phospho-ERK1/2 levels were further increased in cells treated with carboplatin in both flow and static culture groups (Figure 5B). ERK1/2 activity is modulated by the upstream kinase MEK (Figure 5C). An upregulation of total MEK levels was observed in cells grown under flow and further increased (Figure 5B) in cultures that were both grown under flow and treated with carboplatin.

Interestingly, total EGFR expression decreased while phospho-ERK increased in cells treated with carboplatin in both static and flow cultures (Figure 5B). This led us to investigate whether ECM-mediated mechano-sensing plays a role in chemoresistance. Previous studies suggest that ERK1/2 activity could also be regulated by focal adhesion complex proteins, such as vinculin, paxillin, and focal adhesion kinase (FAK) [91]. Expression of FAK significantly decreased when cells were treated with carboplatin in static cultures and was nearly undetectable in cultures grown under flow and treated with carboplatin. Phospho-FAK levels concomitantly were undetectable in all groups except static untreated cultures. Paxillin and vinculin are among the other proteins that constitute the focal adhesion complex. Both paxillin and vinculin were downregulated under flow-induced shear stress and expression further decreased when the respective cultures were treated with carboplatin (Figure 5B).

### 3.6. Photoimmunotherapy Is Equally Effective in Reducing Tumor Area Under Static and Flow-Induced Shear Stress Conditions

**Figure 6:**
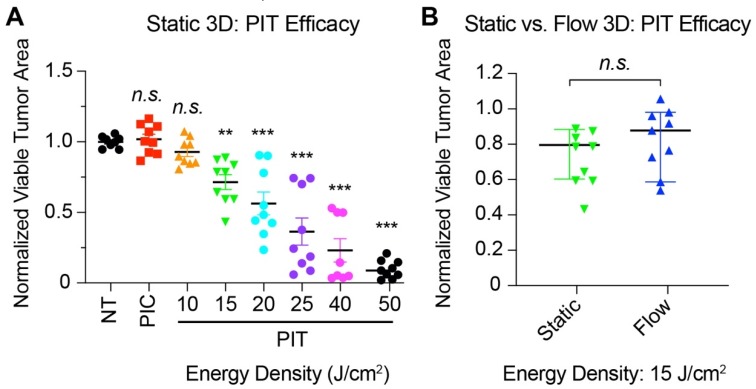
PIT efficacy in 3D tumors. (**A**) Dose-dependent change in normalized viable tumor area in static 3D cultures treated with PIC (1 μM BPD equivalent) and increasing energy densities (10–50 J/cm^2^ @ 50 mW/cm^2^). Significant tumoricidal efficacy is observed in a light-dose-dependent manner, starting at 15 J/cm^2^. (One-way ANOVA with Dunnett’s post hoc test; n.s., not significant; ** *p* < 0.01, *** *p* < 0.001, N = 9) (**B**) Comparison of cytotoxic response in PIT-treated 3D cultures under static and flow conditions. For quantitative analysis of fluorescence images, a minimum nodule size cut-off of 2000 µm^2^ (clusters of ~15–20 cells) was used to establish normalized viable tumor area. PIT is equally effective in 3D tumors grown in static cultures (green) and under flow-induced shear stress (blue) (in contrast to flow-induced chemo-resistance shown in Figure 4) (Two-tailed t test; n.s., not significant; N = 9).

Photochemical targeting of tumors has been shown to be effective in a variety of cancer types, including ovarian cancer. Here, a low-dose approach is leveraged to target EGFR overexpression in tumors grown under flow-induced shear stress (Figure 6). This approach builds on an extensive body of published studies [49,59,60,61,67,68,69,70,94,95,96,97], demonstrating the ability of photodynamic therapy (PDT) and a targeted variant, photoimmunotherapy (PIT), to selectively induce photodamage and induce cytotoxicity in a manner that is mechanistically-distinct from traditional chemotherapy. Whether this approach is effective in targeting molecular and phenotypic changes in 3D ovarian tumors grown under flow-induced shear stress was investigated for the first time in this study. A well-established anti-EGFR PIC [49,60,61,67,68,69,70] was used to target and deliver a photosensitizing agent, in this case, BPD, to 3D ovarian tumors. Figure 6A shows normalized viable tumor area in static 3D cultures following incubation with PIC (1 μM BPD equivalent) for 24 h and subsequent irradiation with increasing energy densities ranging from 10–50 J/cm^2^ @ 50 mW/cm^2^. At 25 J/cm^2^ and 50 J/cm^2^, the mean normalized tumor area in static 3D cultures was 0.36 ± 0.09 and 0.09 ± 0.02, respectively (Figure 6A). A significant decrease in normalized viable tumor area was observed at 15 J/cm^2^ for both static 3D cultures and 3D tumors grown under flow-induced shear stress (Figure 6A and Figure S8). A comparison of PIT efficacy in static versus flow conditions is shown in Figure 6B. In contrast to the flow-induced resistance to carboplatin shown in Figure 4B, low-dose PIT (15 J/cm^2^ @ 50 mW/cm^2^) was equally effective in reducing tumor area in cultures grown under flow-induced shear stress (mean normalized tumor area: 0.81 ± 0.06) compared to static cultures (Figure 6B) (mean normalized tumor area: 0.72 ± 0.05) (*p* > 0.05).

## 4. Discussion

Approximately 38% of all malignant ascites in females result from epithelial ovarian cancer, the most common primary site associated with ascites [3,5,98,99]. Approximately one-third of all ovarian cancer patients will develop ascites, but this number can be as high as nearly 90% [99] in patients with advanced-stage disease (stages III or IV). The presence and progression of ascites are associated with grim survival statistics, a decreased quality of life, and may have predictive value for malignant ovarian tumors [3,5,11,14,15,16,17,26,31,99,100,101,102]. The predictive value of ascites remains a source of debate, as is the case with most clinical and biological markers for this challenging disease [98,103,104,105]. Ascites in ovarian cancer patients is treated by managing the underlying disease, typically via surgical resection and adjuvant platinum-based chemotherapy. Unfortunately, an estimated 85% of ovarian cancer patients who achieve remission following first-line therapy will develop recurrent disease, which is associated with a median survival of 12 to 24 months [2,12,14,105]. Intractable ascites often develop in these patients, a major co-morbidity that is managed by draining the fluid to temporarily alleviate the associated pain and discomfort [3,5,14,103,104]. Treatment approaches to reduce ascites and improve chemotherapy efficacy include the use of anti-vascular endothelial growth factor agents as well as targeting the renin-angiotensin system [3,5,14]. These efforts have shown promise in preclinical studies and trials, but this remains an area that requires further investigation.

The effect of malignant ascites on the growth and progression of ovarian cancer cells has been primarily studied in the context of the cellular and acellular factors present in ascitic fluid [3,5,11,14,15,16,17,26,31,99,100,101,102]. The cellular components of ascites include tumor and stromal cells present as single cells or as tumor clusters/spheroids. A complex milieu of proteins, cytokines, metabolites, and exosomes, among other factors, constitute the acellular components of ascitic fluid [3,5,11,14,15,16,17,26,31,99,100,101,102]. Interaction with ascites has been shown to modulate the molecular phenotype of ovarian cancer cells and to contribute to tumor heterogeneity [11,16,17,31,106]. Ahmed and colleagues evaluated the effect of patient-derived ascites on integrin-mediated modulation of proliferation and function in a human ovarian surface epithelial (HOSE) cell line and 4 ovarian cancer cell lines, with varying degrees of invasiveness [31]. An ascites-induced increase in the expression of α6 integrin was observed in the four ovarian cancer cell lines with no change in HOSE cells. Similarly, an ascites-induced increase in adhesion and proliferation was observed in the ovarian cancer cell lines, with no change in the HOSE cells. In the presence of ascites, increased expression of urokinase plasminogen activator (uPA), a serine protease involved in the metastatic cascade [107], was observed in more invasive ovarian cancer cell lines. No change was seen in HOSE cells and non-invasive ovarian cancer cells [31]. Ascites-induced increased expression of α6 integrin and uPA also correlated with activation of Ras and ERK, as well as with increased invasiveness in the invasive ovarian cancer cell lines [31]. A related effort focused on the isolation and characterization of ovarian cancer cells from the ascites of patients with chemonaive disease compared to chemoresistant/at recurrence disease [16]. The authors demonstrated the separation of ascites into distinct epithelial/tumorigenic and mesenchymal/non-tumorigenic populations. Interestingly, cells from the ascites of patients with chemoresistant disease were shown to be predominantly epithelial with a tendency to express mRNA associated with cancer stem cell markers [16]. A follow-up study characterized the proteome signatures of ovarian cancer cells derived from ascites of patients with chemonaive disease and in the same patients at the time of recurrence [17]. The study identified 353 proteins that were differentially expressed, corresponding to a range of candidate pathways including cytoskeleton rearrangement, cell-cell adhesion, and cell transport. The role of flow-induced shear stress on these valuable insights remains to be elucidated.

Flow-induced shear stress has been shown to induce changes in the morphologic, genetic, and protein profiles of cancers, including ovarian cancer [18,19,20,21,22,23,24,25,26]. Previous computational simulations of gastrointestinal models estimated flow-induced shear stress in the peritoneum in the range of 0.14 dyne/cm^2^ to 11 dyne/cm^2^ [108]. Using ^I25^l-labeled human serum albumin tracers, Nagy et al. showed a dramatic increase in the rate of peritoneal fluid appearance up to 3 to 5 µL/min in animals bearing ascites, compared to the control animals without ascites (0.06–0.08 µL/min) [109]. We have previously shown that physiologically-relevant flow-induced shear stress (3 dyne/cm^2^, 2 µl/min) induces a post-translational upregulation and activation of the EGFR in perfusion models of 3D ovarian cancer [25]. Flow-driven EGFR upregulation is likely a result of decreased receptor degradation and increased recycling [25]. A concomitant, transcriptionally-regulated, flow-induced decrease in E-cadherin, along with an increase in vimentin expression, was observed [25]. These changes in E-cadherin and vimentin expression, along with altered tumor morphology, are consistent with epithelial-mesenchymal transition (EMT). EMT is a phenotypic transition that is often associated with increased resistance to targeted biologics and chemotherapy [25,110,111]. Two critical members of the EGFR signaling pathway are MEK and ERK, which play an important role in cancer progression and chemoresistance [112,113,114]. The current study shows, for the first time, the impact of flow-induced shear stress on resistance to carboplatin and modulation of EGFR-mediated survival pathways in adherent 3D ovarian tumors.

Transitioning from a chemo-sensitive phenotype in static culture to a chemoresistant phenotype under continuous flow is associated with changes in multiple signaling cascades involved in morphological changes and cell survival. We have previously reported that expression of the EGFR, a poor prognostic factor of ovarian cancer, is post-translationally upregulated under flow-induced shear stress [25]. EGFR has been shown to modulate the ERK-pathway in many cancer types, including ovarian cancer. ERK is critical for cell survival and is activated by multiple extracellular and intracellular factors. Using a perfusion model, we found that EKR1/2 phosphorylation was significantly upregulated in ovarian cancer cells under flow-induced shear stress (0.3 Pa or 3 dyne/cm^2^) [25] as well as when treated with carboplatin (500 μM). Our findings are consistent with previous studies that observed elevated phospho-ERK1/2 levels in endothelial cells [115,116] and preosteoblast cells [117] in response to flow-induced shear stress (1–10 dyne/cm^2^). A number of studies have also indicated that platinum agents can induce ERK activation in ovarian cancer and other cancers [118,119,120,121,122,123,124,125]. However, whether the elevation of phospho-ERK1/2 contributes to or prevents certain death mechanisms, such as apoptosis, remains controversial.

Previous studies have reported that EGFR is overexpressed and phospho-ERK1/2 is elevated in 35–70% of human ovarian cancer samples [126,127]. Combinations with ERK/EGFR inhibitors and chemotherapy hold promise for synergistic anti-cancer effects. Hayakawa et al. reported that inhibition of the ERK pathway sensitized ovarian cancer cells to cisplatin [119]. However, a clinical trial with cetuximab in combination with carboplatin exhibited only modest anti-cancer efficacy in patients with ovarian cancer [128]. Potential barriers to cetuximab efficacy include stochastic interference from high EGFR density induced by flow-induced shear stress and the inability to saturate the EGFR (i.e., sub-optimal antibody delivery efficacy). It is also possible that the high mutational burden commonly seen in ovarian cancer leads to constitutively active ERK, which may not respond to EGFR blockade upstream of ERK. In this study, EGFR expression was downregulated in cells treated with carboplatin, while phospho-ERK expression was elevated (Figure 5). To investigate the upstream regulator of ERK, we confirmed that MEK, which phosphorylates and activates ERK, is also upregulated in OVCAR-5 cells under flow and treated with carboplatin.

These findings directed us to look for other mechanisms and cross-talk between pathways that could activate ERK and lead to chemoresistance. We previously reported that E-cadherin is downregulated in OVCAR-5 cells under flow-induced shear stress [25]. Depletion of E-cadherin is thought to be the hallmark of EMT and metastasis. Interestingly, loss of E-cadherin was reported to activate ERK1/2 phosphorylation in lung cancer patients and was associated with a disseminated phenotype of cancer [129]. Consistent with our findings, E-cadherin was also associated with activation of the MAPK pathway through EGFR in a ligand-independent manner [130]. Previous studies have also shown that ERK can be activated by signaling proteins (i.e., vinculin, paxillin, FAK) at the focal adhesions. Vinculin has been shown to phosphorylate ERK in a manner that requires interaction between FAK and paxillin [91]. Cancer cells that lose expression of vinculin show enhanced metastasis [131,132] as well as resistance to apoptosis [91]. The absence of vinculin increases the interaction of paxillin and FAK, which in turn enhances phosphorylation of ERK1/2 [91]. In our study, we showed that flow-induced shear stress upregulates phospho-ERK and downregulates vinculin, phospho-paxillin, and phospho-FAK. These results agree with others, showing that flow-induced shear stress can activate ERK in a FAK-dependent or FAK-independent manner [133]. However, the FAK-independent pathway that is involved in the activation of ERK remains largely unknown and warrants further investigation. Others have suggested that phosphorylation of paxillin could contribute to platinum-agent resistance via ERK activation [134]. In this study, we found that carboplatin downregulates p-paxillin, vinculin, and p-FAK under both static and flow conditions. These results suggest that the observed carboplatin resistance under flow conditions in OVCAR-5 cells is likely to be independent of the signaling proteins at the focal adhesions.

PDT uses visible light to electronically excite a PS to generate RMS that are toxic to nearby targets [48,135,136]. The spatial and temporal control inherent to PDT has been leveraged to target various tumor compartments: (i) non-cellular stromal components (e.g., hyaluronic acid and collagen), (ii) stromal cells (e.g., fibroblasts), or (iii) tumor cells directly [137]. A limited set of studies have assessed PDT-related regimens in the context of physical stress in the tumor microenvironment [137,138]. PDT can help decrease solid stress and, thereby, modulate microenvironmental factors that influence tumor growth and treatment response, such as hypoxia and interstitial fluid pressure [139,140,141,142,143,144]. To improve selective delivery, the PS can be attached to a variety of targeting moieties, such as carbohydrates, peptides, folic acid and antibodies, or nanocarriers that target markers that are overexpressed on cancer cells [48,97,136,145,146,147,148,149]. PIT involves the use of an antibody-based PIC to enhance PS selectivity and has been shown to be effective in a variety of cancer types, including ovarian cancer [49,60,61,67,68,69,70,94,95,96,97]. The efficacy of anti-EGFR targeted PIT in adherent 3D tumors grown under flow-induced shear stress was investigated for the first time in this proposal.

Future studies will evaluate the implications of the findings presented here in additional cell lines and tumor samples and in the context of rationally-designed combinations. Previous studies from our groups and others [49,60,61,67,68,69,70,150] will be leveraged, demonstrating the ability of PDT and PIT to potentiate the efficacy of platinum-based chemotherapy, reduce the number of chemotherapy cycles, reverse platinum-based resistance, and elicit an anti-tumor immune response.

## Supplementary Materials

The following are available online at https://www.mdpi.com/2077-0383/9/4/924/s1, **Figure S1.** Components, dimensions, assembly and imaging of a perfusion model for ovarian cancer. **Figure S2.** Size distributions of 3D tumors in static cultures following carboplatin treatment. **Figure S3.** Carboplatin efficacy in static 3D cultures following quantitative image analysis for a range of minimum nodule size cut-offs. **Figure S4.** Representative images of carboplatin-treated 3D tumors in static cultures following quantitative image analysis for a range of minimum nodule size cut-offs. **Figure S5.** Quantitative image processing in a perfusion model for 3D ovarian cancer using customized analysis routines. **Figure S6.** Carboplatin efficacy in 3D ovarian tumors grown under flow-induced shear stress following quantitative image analysis for a range of minimum nodule size cut-offs. **Figure S7.** Comparison of normalized viable tumor areas in 3D ovarian tumors grown in static cultures or under flow-induced shear stress following quantitative image analysis for a range of minimum nodule size cut-offs. **Figure S8.** PIT efficacy, relative to respective no treatment controls, in 3D ovarian tumors grown in static cultures or under flow-induced shear stress.
