# Supplementary material for: Flow-induced Shear Stress Confers Resistance to Carboplatin in an Adherent Three-Dimensional Model for Ovarian Cancer: A Role for EGFR-Targeted Photoimmunotherapy Informed by Physical Stress
Source: J Clin Med. 2020 Mar 28;9(4):924. doi: 10.3390/jcm9040924 (PMC7230263; doi:10.3390/jcm9040924)
Supplement: Supplementary file 1 [file jcm-09-00924-s001.pdf]

Supplementary Materials:

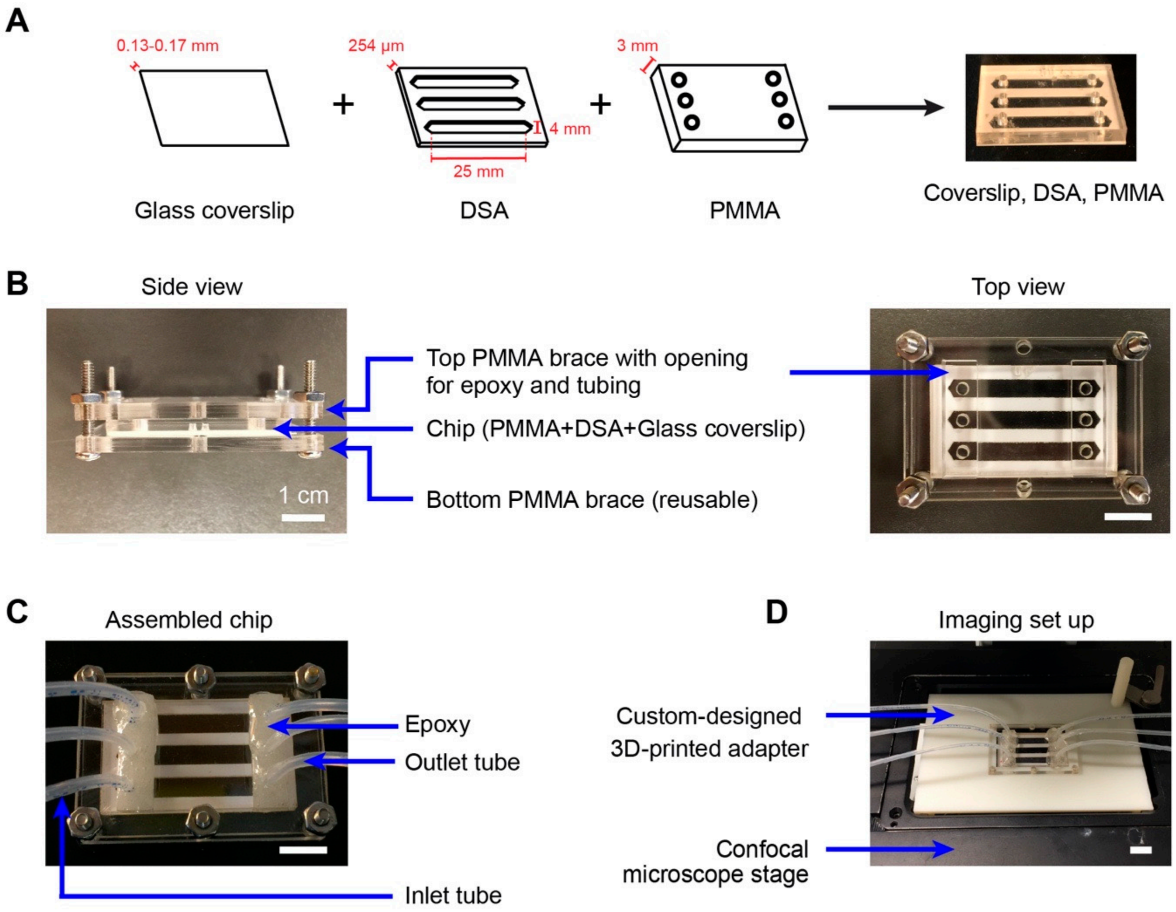

Figure S1. Components, dimensions, assembly, and implementation of the perfusion model. **(A)** The perfusion model is constructed from three major components: (i) a glass coverslip, (ii) a layer of micromachined double-sided adhesive (DSA) with embedded channels, and (iii) a polymethylmethacrylate (PMMA) layer with micromachined inlet and outlet ports. **(B)** To enable extended culturing of cells under flow, the basic chip design in (A) is placed between two PMMA braces. The bottom PMMA brace is reusable. The top PMMA brace has cutouts that, in the assembled chip **(C)**, hold the inlet and outlet tubing with epoxy. **(D)** A 3D-printed adapter fitted for the confocal microscope stage and perfusion chamber were among the tools that enabled full-chip fluorescence imaging in x, y, and z. Scale bars: 1 cm.

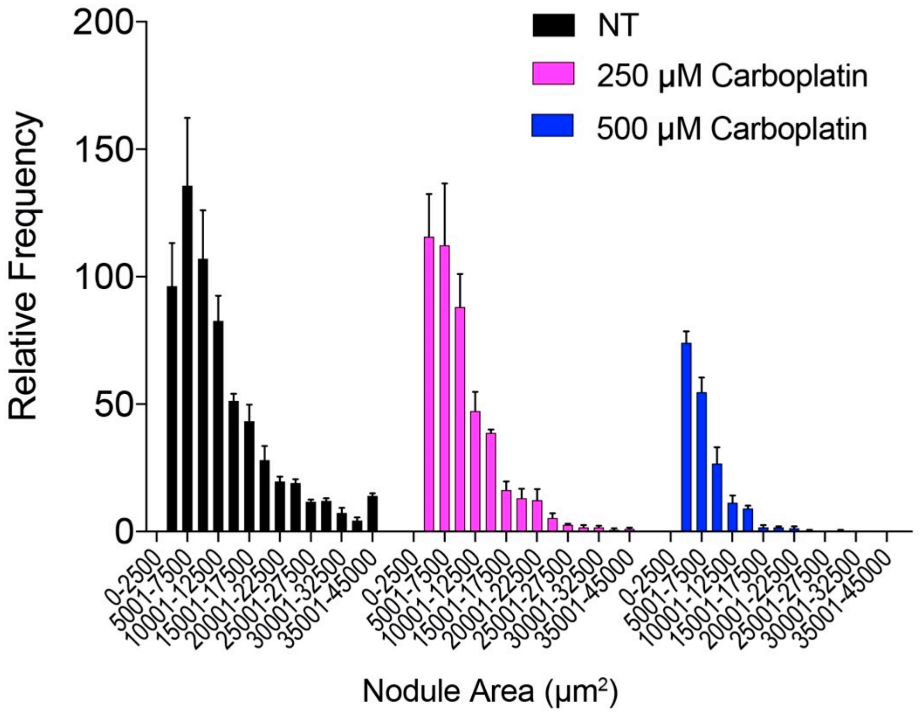

Figure S2. Relative frequency and size distributions of adherent 3D OVCAR-5 ovarian tumors following carboplatin treatment in static cultures at 250 μM and 500 μM. Similar to previous studies, the dose-dependent reduction in the live area is similar across all sizes of 3D nodules.

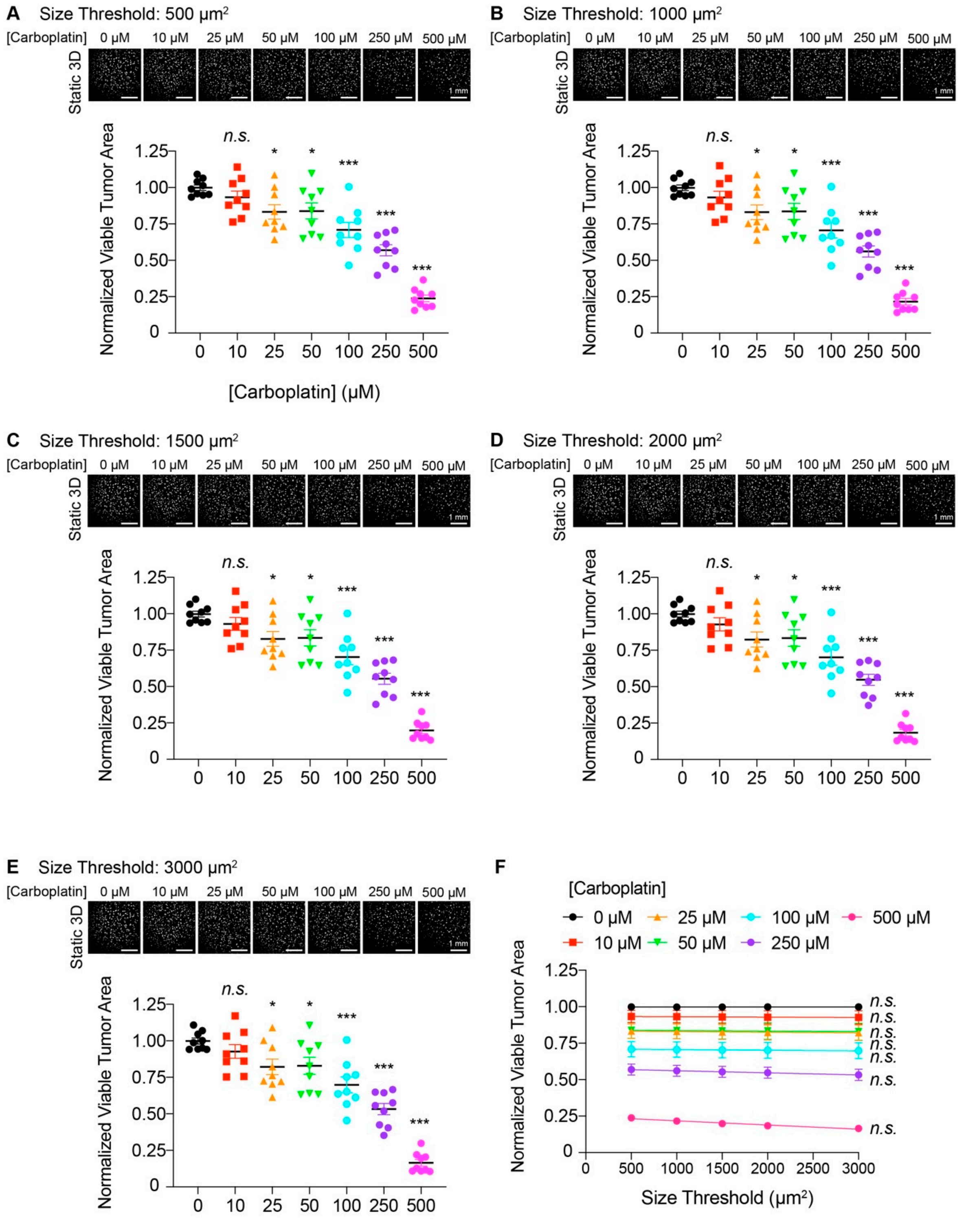

Figure S3. (A-E) Carboplatin dose response in static 3D cultures analyzed using minimum nodule size cut-offs ranging from 500 to 3000 μm². (n.s. not significant, *p<0.05; ***p<0.001; N=9) **(F)** A comparison of normalized viable tumor areas for the range of carboplatin doses in (A-E), analyzed using minimum nodule size cut-offs ranging from 500 to 3000 μm², showing no significant difference in the dose response curves (ANOVA, P > 0.05, n.s. not significant). Scale bars: 1 mm.

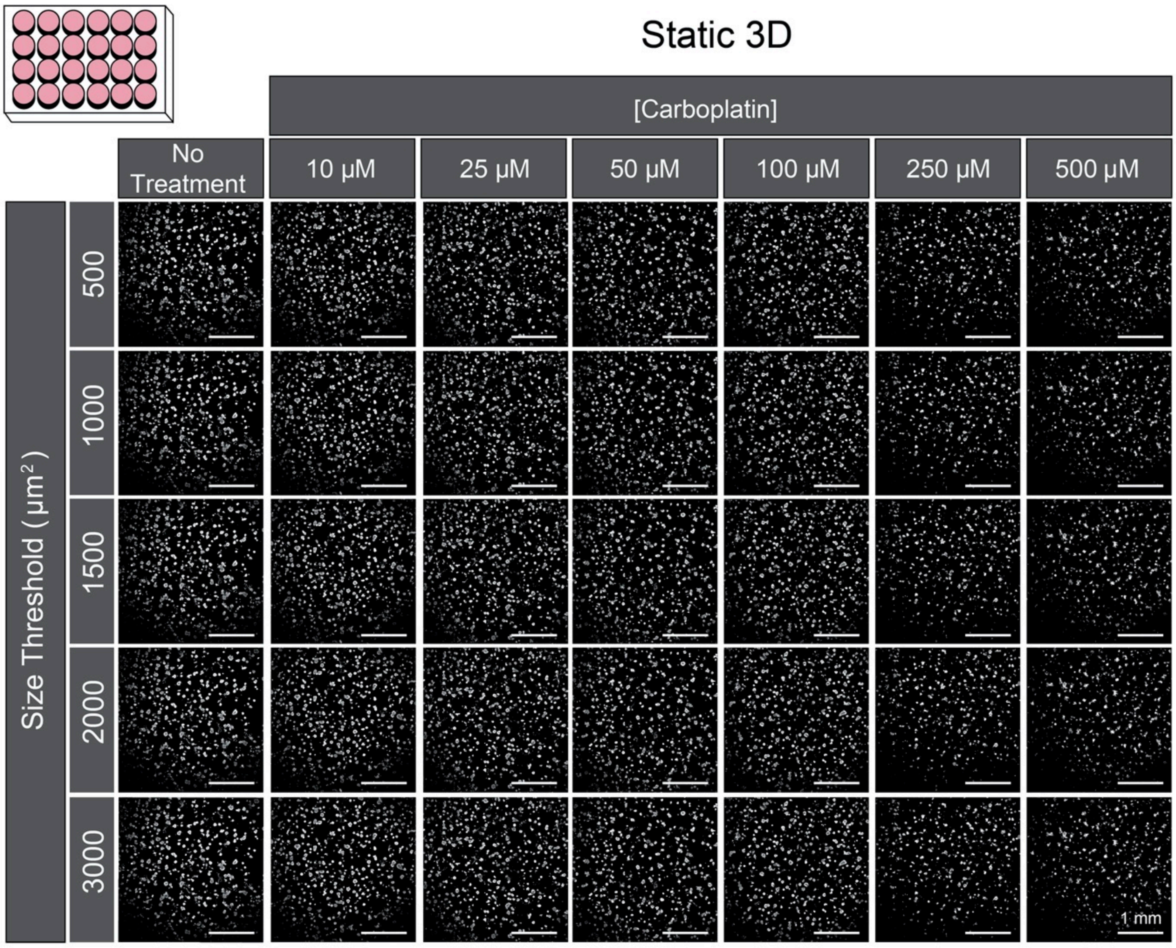

Figure S4. Side-by-Side Comparison of the confocal images of 3D OVCAR-5 tumor nodules grown under static condition at different carboplatin concentrations (0-500 μm) and with different size thresholds for data analysis. Scale bars: 1 mm.

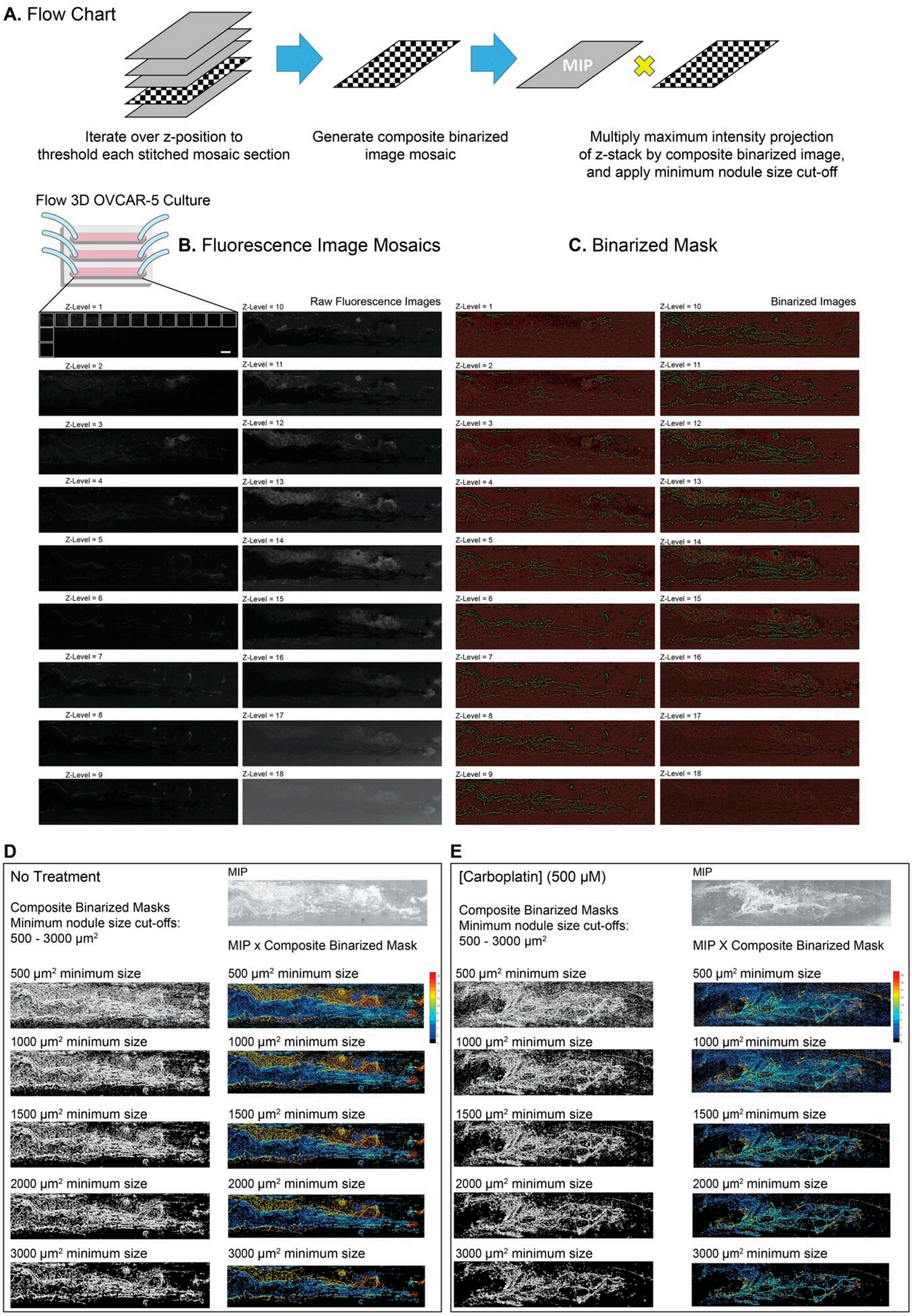

Figure S5. (A) Workflow for analysis of confocal multi-area mosaic z-stacks of calcein stained full flow channels. Individual image fields are stitched to form full area mosaics for each z-position. These were segmented then projected to form a composite binarized mosaic which is multiplied by the maximum intensity projection (MIP) to form a single flattened image for use in subsequent analysis. Representative calcein fluorescence image mosaic z-stacks are shown in **(B)** and corresponding binarized masks for each z-section in **(C)**. Application of this

analysis is shown for an untreated flow culture in **(D)** and carboplatin-treated culture in **(E)**. In **(D)** and **(E)**, the effectiveness of minimum nodule size cut-off in image processing is shown with composite binarized masks on the left and the MIP times mask product on the right corresponding to each minimum nodule size cut-off evaluated from 500 μm^2 to 3000 μm^2. In MIP-mask product images in **(D)** and **(E)**, pixel intensity is displayed using a multi-color look up table for visual contrast.

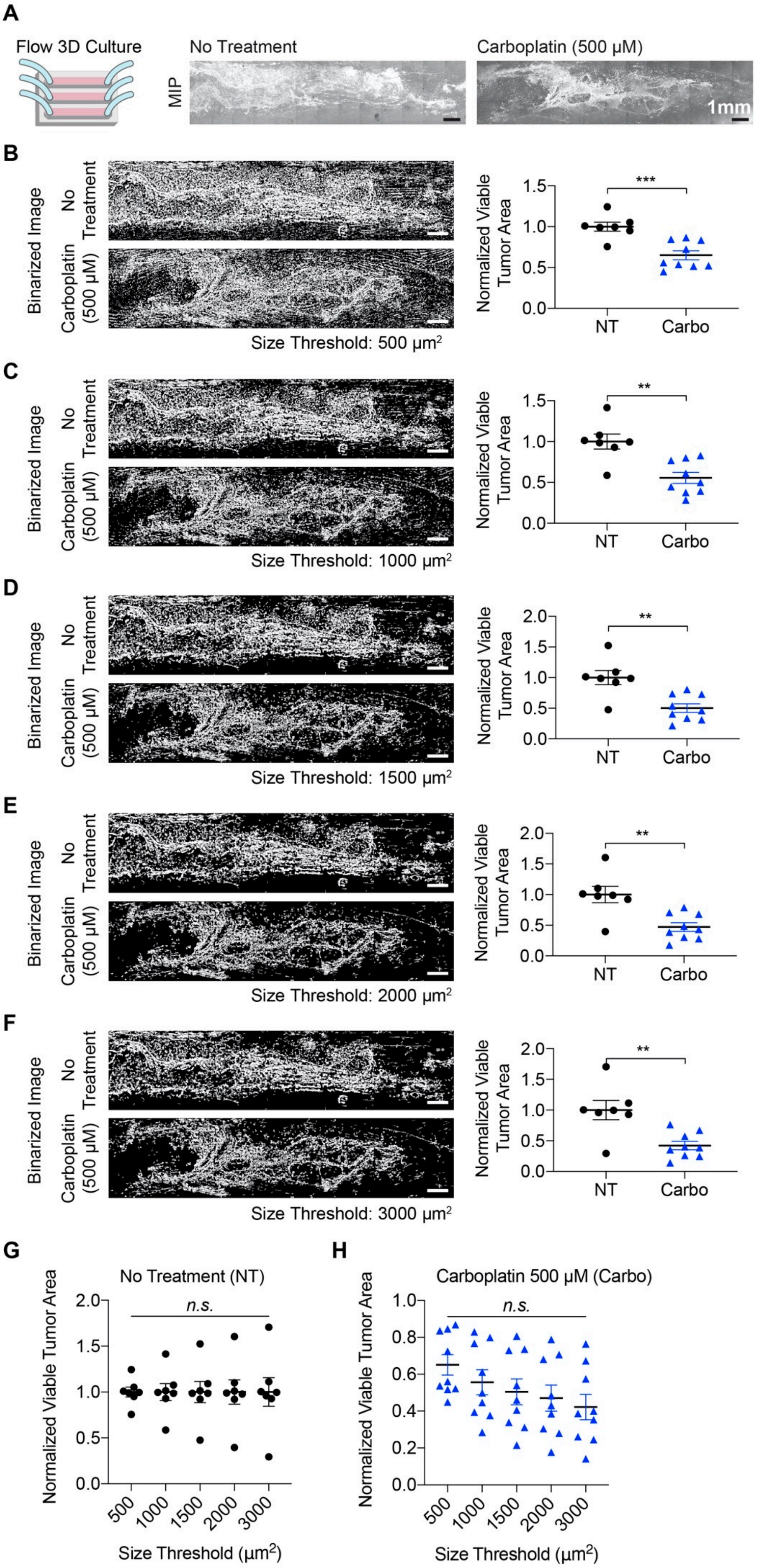
A
Flow 3D Culture
No Treatment
Carboplatin (500 µM)
MIP
1mm
B
Binarized Image
No Treatment
Carboplatin (500 µM)
Size Threshold: 500 µm²
Normalized Viable Tumor Area
***
NT
Carbo
C
Size Threshold: 1000 µm²
**
D
Size Threshold: 1500 µm²
E
Size Threshold: 2000 µm²
F
Size Threshold: 3000 µm²
G
No Treatment (NT)
n.s.
Size Threshold (µm²)
H
Carboplatin 500 µM (Carbo)

Figure S6. (A-F) Normalized viable tumor areas in 3D ovarian tumors grown under flow, treated with 500 μM carboplatin, and analyzed using minimum nodule size cut-offs ranging from 500 to 3000 μm^2. (**p<0.01; ***p<0.001; N=9) **(G-H)** A comparison of no treatment controls **(G)** and carboplatin treated cultures **(H)** (from A-F), showing no significant difference in normalized viable tumor areas for minimum nodule size cut-offs ranging from 500 to 3000 μm^2 (ANOVA, P > 0.05, n.s. not significant). Scale bars: 1 mm.

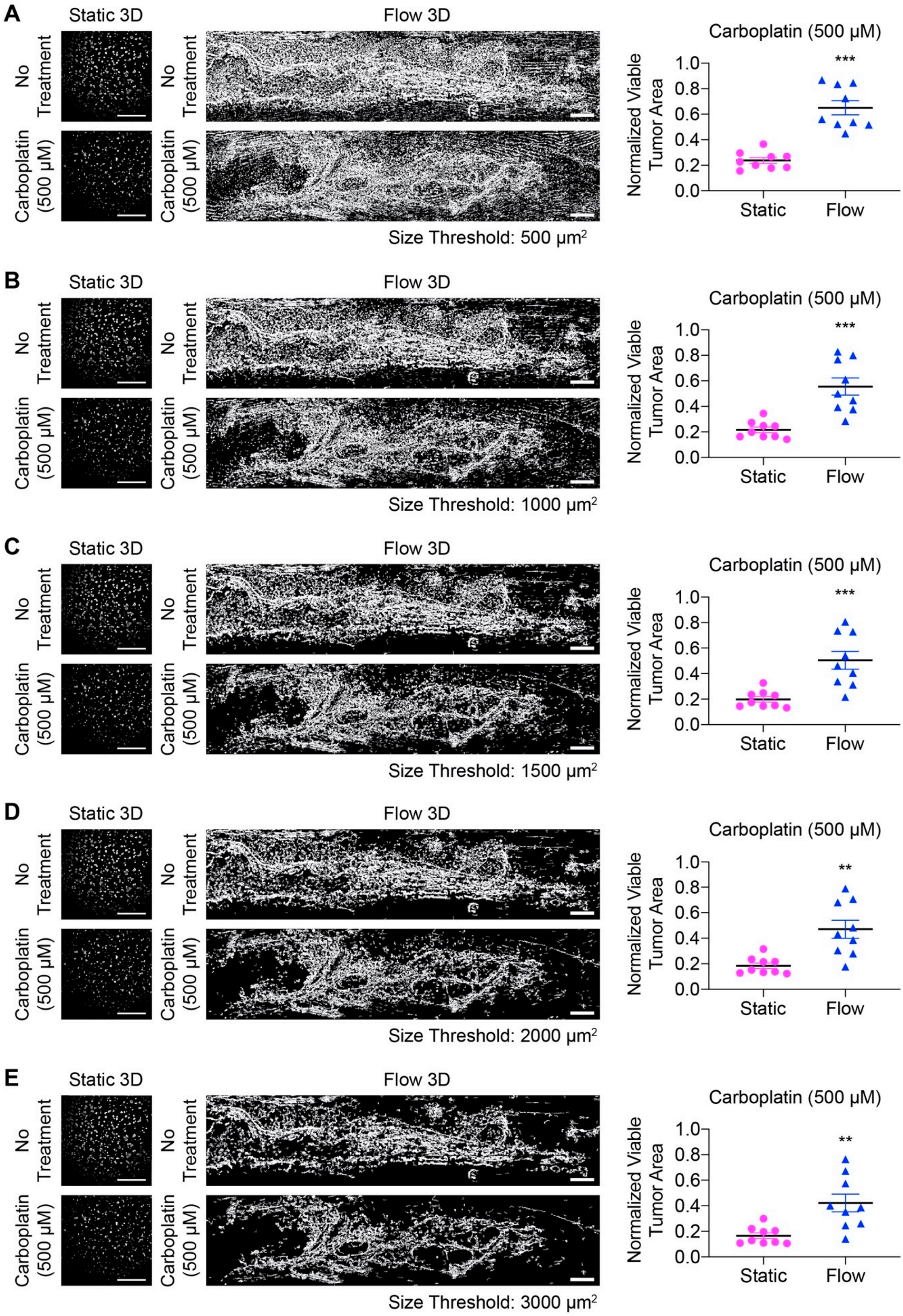

Figure S7. Comparison of normalized viable tumor areas in 3D ovarian tumors grown under static and flow condition. 3D ovarian tumors were treated with 500 μM carboplatin and analyzed using minimum nodule size cut-offs ranging from 500 to 3000 μm^2. (Two-tailed t test; **p<0.01, ***p<0.001; N=9) Scale bars: 1 mm.

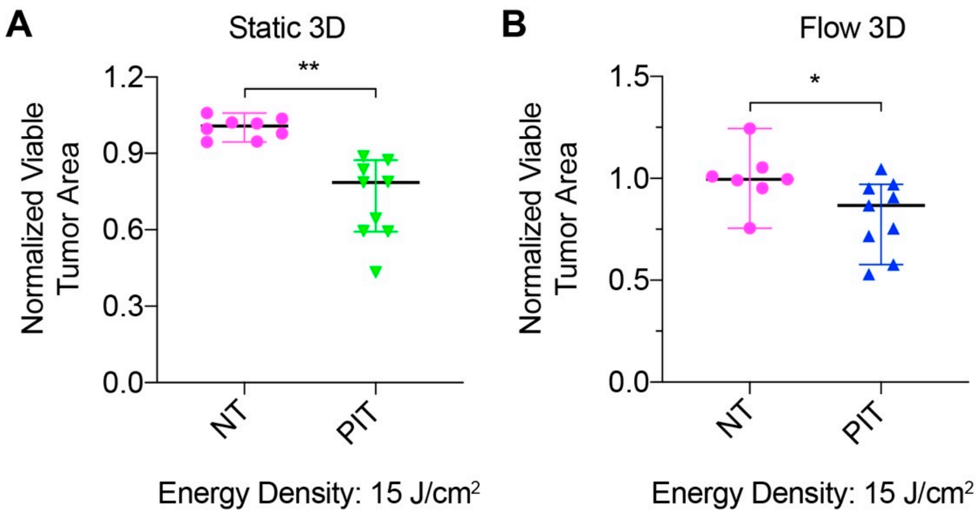

Figure S8. PIT with 1 mM BPD equivalent and an energy density of 15 J/cm[2] results in a significant reduction in normalized viable tumor area in both **(A)** static 3D cultures and **(B)** 3D cultures grown under flow-induced shear stress (Two-tailed t test; *p<0.05, **p<0.01; N=9).
